# Supplementary material for: Graphdiyne and Nitrogen-Doped Graphdiyne Nanotubes as Highly Efficient Electrocatalysts for Oxygen Reduction Reaction
Source: Int J Mol Sci. 2023 Nov 27;24(23):16813. doi: 10.3390/ijms242316813 (PMC10706831; doi:10.3390/ijms242316813)
Supplement: Supplementary file 1 [file ijms-24-16813-s001.zip › ijms-2714406-supplementary.pdf]

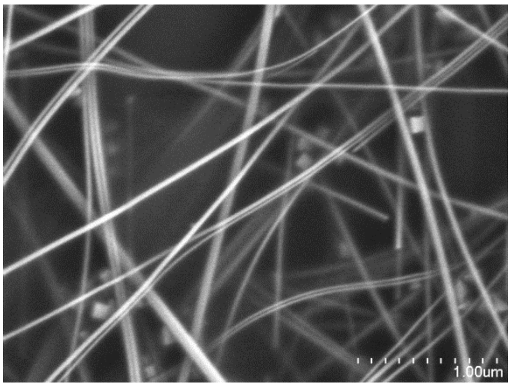

Figure S1 SEM image of Cu nanowire.

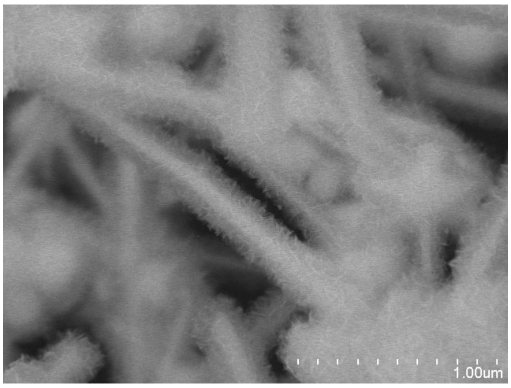

Figure S2 SEM image of GDY@Cu.

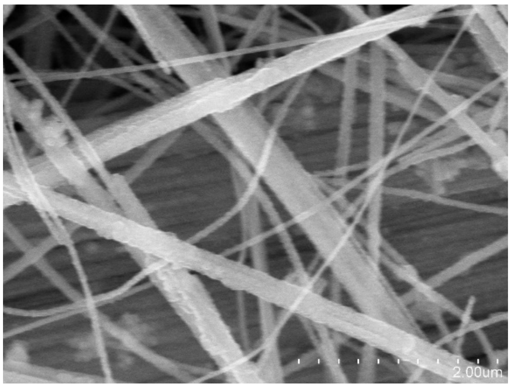

Figure S3 SEM image of NGDY@Cu.

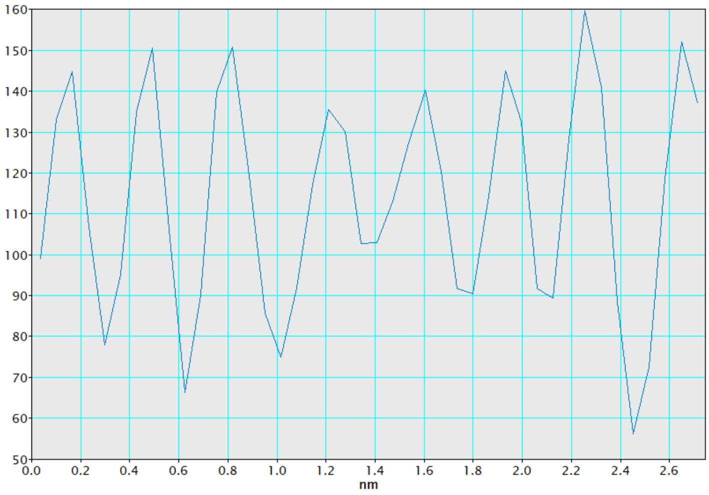

Figure S4 The interlayer distance of GDY nanotube.

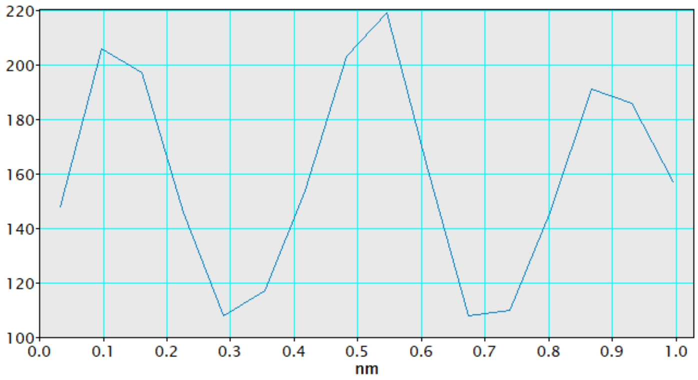

Figure S5 The interlayer distance of NGDY nanotube.

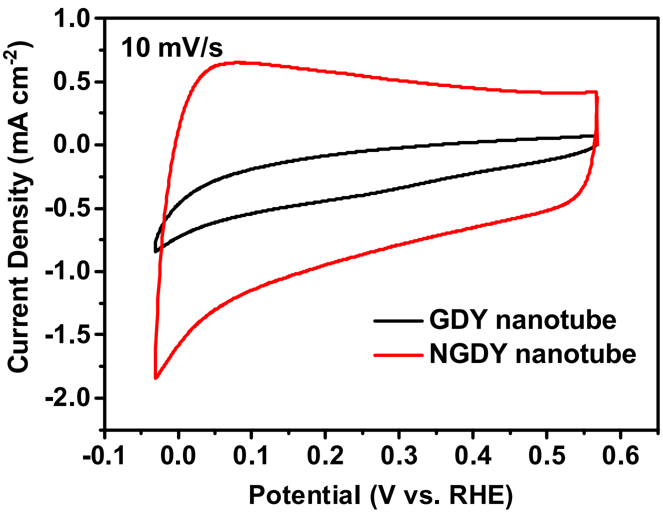

Figure S6 CV curves of GDY nanotube and NGDY nanotube.

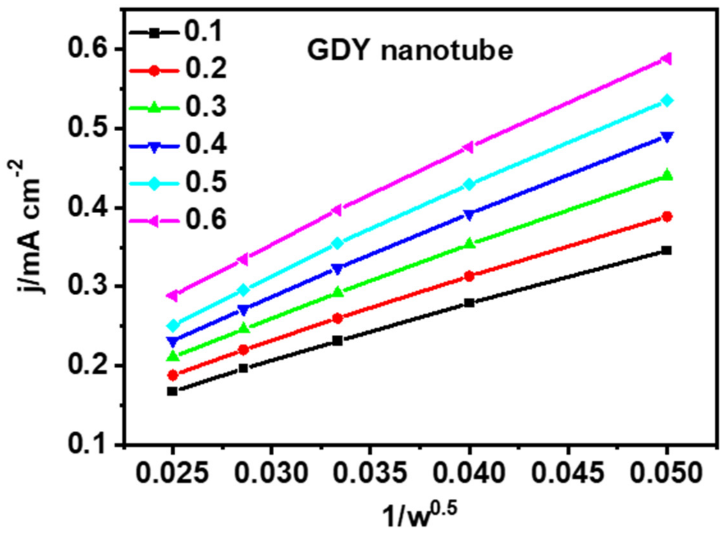

Figure S7 Koutecky-Levich plots of GDY.

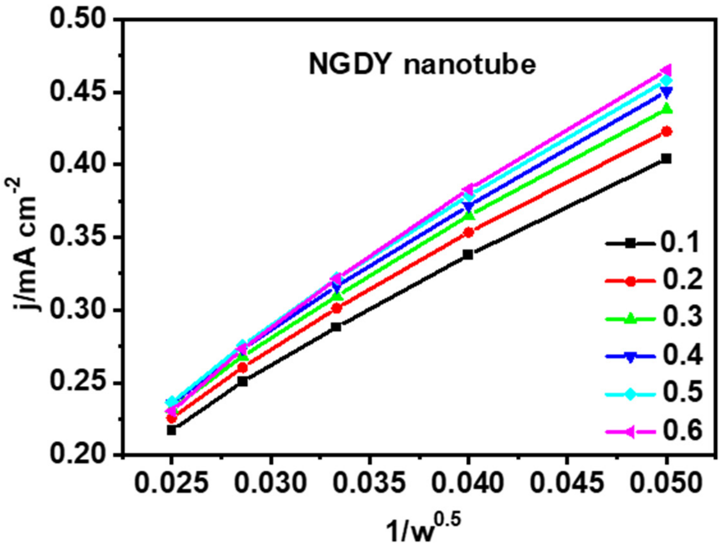

Figure S8 Koutecky-Levich plots of NGDY.

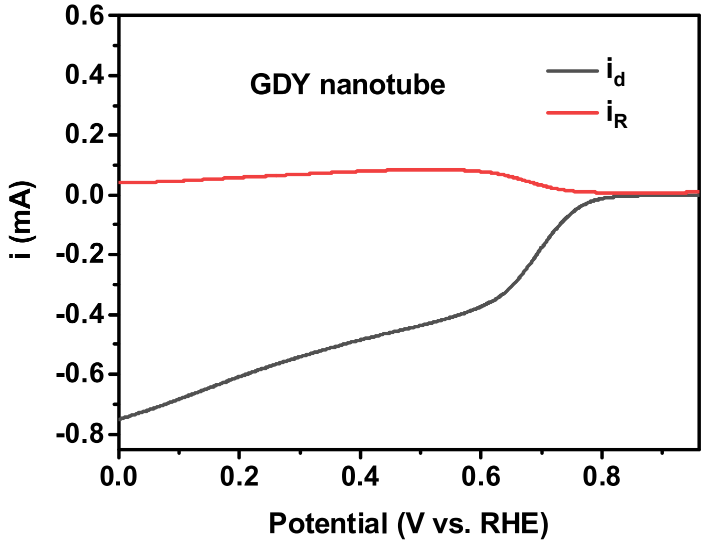

Figure S9 RRDE linear sweep voltammogram of GDY.

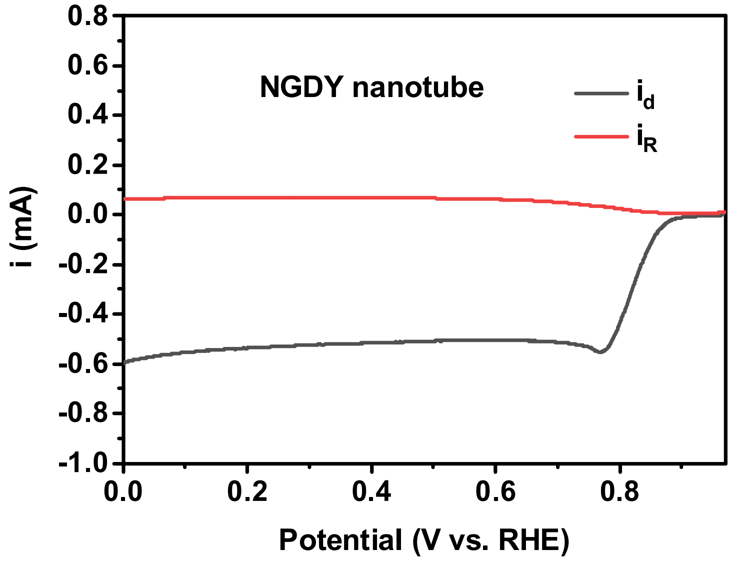

Figure S10 RRDE linear sweep voltammogram of NGDY.

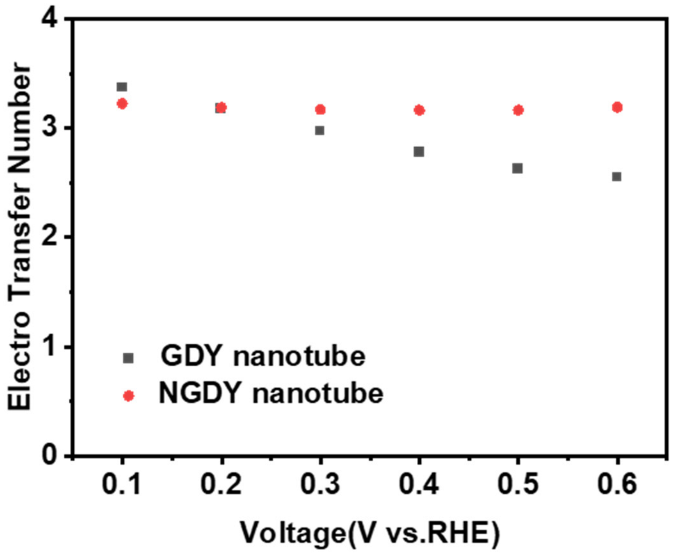

Figure S11 Electron transfer numbers of GDY nanotube and NGDY nanotube electrodes according to RRDE.

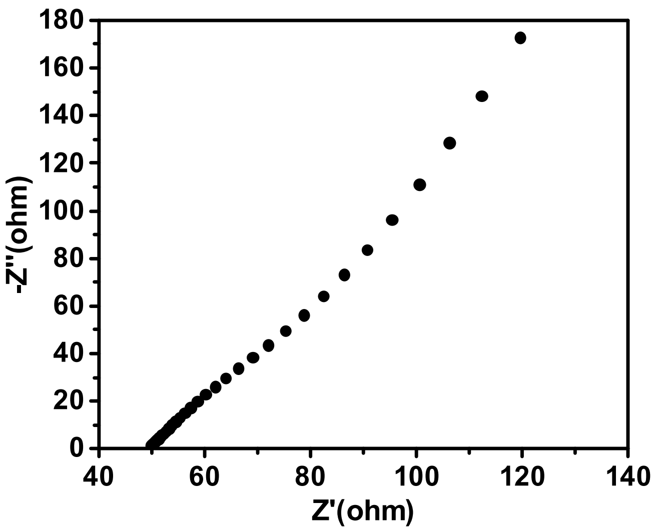

Figure S12 Nyquist plots of solution resistance.
